# Plasma tsRNAs as novel diagnostic biomarkers for renal cell carcinoma

**DOI:** 10.1002/ctm2.1575

**Published:** 2024-02-15

**Authors:** Meng Ding, Wanqing Zhou, Wenyuan Chen, Wenjing Mo, Xinyue Guo, Yuhang Li, Changwei Ji, Guangxiang Liu, Wenli Diao, Hongqian Guo

**Affiliations:** ^1^ Department of Urology Nanjing Drum Tower Hospital The Affiliated Hospital of Nanjing University Medical School Institute of Urology Nanjing University Nanjing China; ^2^ Department of Laboratory Medicine, Nanjing Drum Tower Hospital, The Affiliated Hospital of Nanjing University Medical School Nanjing University Nanjing China; ^3^ Department of Urology Nanjing Drum Tower Hospital Clinical College of Nanjing University of Chinese Medicine Nanjing China

Dear Editor,

Early diagnosis greatly benefits renal cell carcinoma (RCC) patients with remarkable higher survival.^1^ Whereas, no appropriate liquid‐biopsy biomarkers has been applied to RCC diagnosis in clinic so far.^2^ Transfer RNA‐derived small RNAs (tsRNAs), a class of newly discovered noncoding RNAs, are stable and abundant in circulation, promising in noninvasive early diagnosis of cancer.^3^, ^4^ Here, we explore the diagnosis values and biological functions of plasma tsRNAs in RCC for the first time.

The study was evaluated and authorised by the Ethics Committee of Nanjing Drum Tower Hospital (2021‐582‐01). The research route diagram was shown in **Figure** **S1**. First, to explore the distinct plasma tsRNA expression profile in RCC patients, we performed small RNA sequencing using RNA extracted individually from plasma of five RCC patients and five healthy controls. The tsRNA expression profile in plasma of RCC patients were obviously changed (Figure 1A), and among 657 tsRNAs detected, 71 tsRNAs were significantly altered (Figure 1B).

**FIGURE 1:**
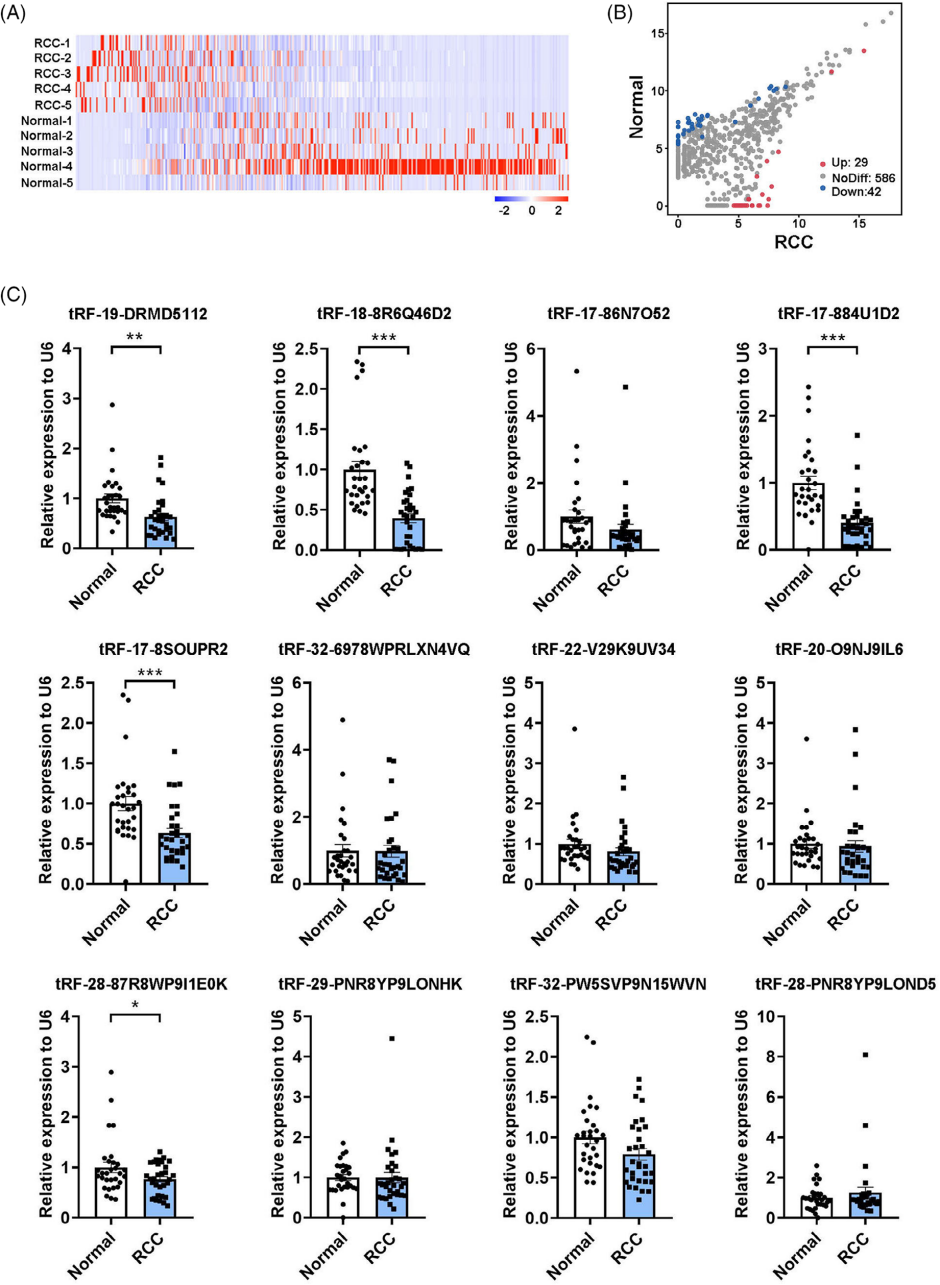
Identification of differentially expressed plasma tsRNAs in RCC patients. (A) Heat map of the expression profile of plasma tsRNAs in 5 RCC patients and 5 healthy controls. (B) Scatter plots of differentially expressed plasma tsRNAs. The red dots indicate upregulated tsRNAs, the blue dots are for downregulated tsRNAs, and the grey dots are for nondifferentially expressed tsRNAs. The grouping criteria is fold‐change ≥ 1.5, *p* < .05. (C) The relative expression levels of 12 plasma tsRNAs in 32 RCC patients and 30 healthy controls using RT‐qPCR. *p < .05; **p < .01; ***p < .001.

Considering the tsRNAs expression levels (CPM > 10) and length (due to the length limitation, the length of tsRNAs should ≥ 17 nt when assessed by RT‐qPCR), we selected 12 tsRNAs for further validation in an independent training set (30 healthy controls and 32 RCC patients) using RT‐qPCR. The detection limits of RT‐qPCR assay using tsRNA specific primers were evaluated by the standard curves developed with corresponding synthetic tsRNA oligonucleotides (**Figure** **S2**, **Table** **S1**). In this training set, 5 out of the 12 tsRNAs (tRF‐19‐DRMD5112, tRF‐18‐8R6Q46D2, tRF‐17‐884U1D2, tRF‐17‐8SOUPR2 and tRF‐28‐87R8WP9I1E0K) were significantly reduced in RCC patients, which was also consistent with the sequencing data (Figure 1C; Tables **S2** and **S3**). Whereafter, the levels of these 5 tsRNAs were further confirmed in another larger validation set (99 healthy controls and 120 RCC patients), and showed a consistent trend (*p* < .0001 for all tsRNAs; Figure 2A and **Table** **S4**). Combining the training and the validation sets for analysis, the results were compatible (*p* < .0001 for all tsRNAs; **Figure** **S3**). Therefore, these data confirmed the stability of the expression pattern. Patient characteristics in the training set and validation set were summarised in **Table** **S5**. Plasma tRF‐28‐87R8WP9I1E0K level was decreased in patients with higher TNM grading (**Table** **S6**), suggesting its potential biological function in RCC progression.

**FIGURE 2:**
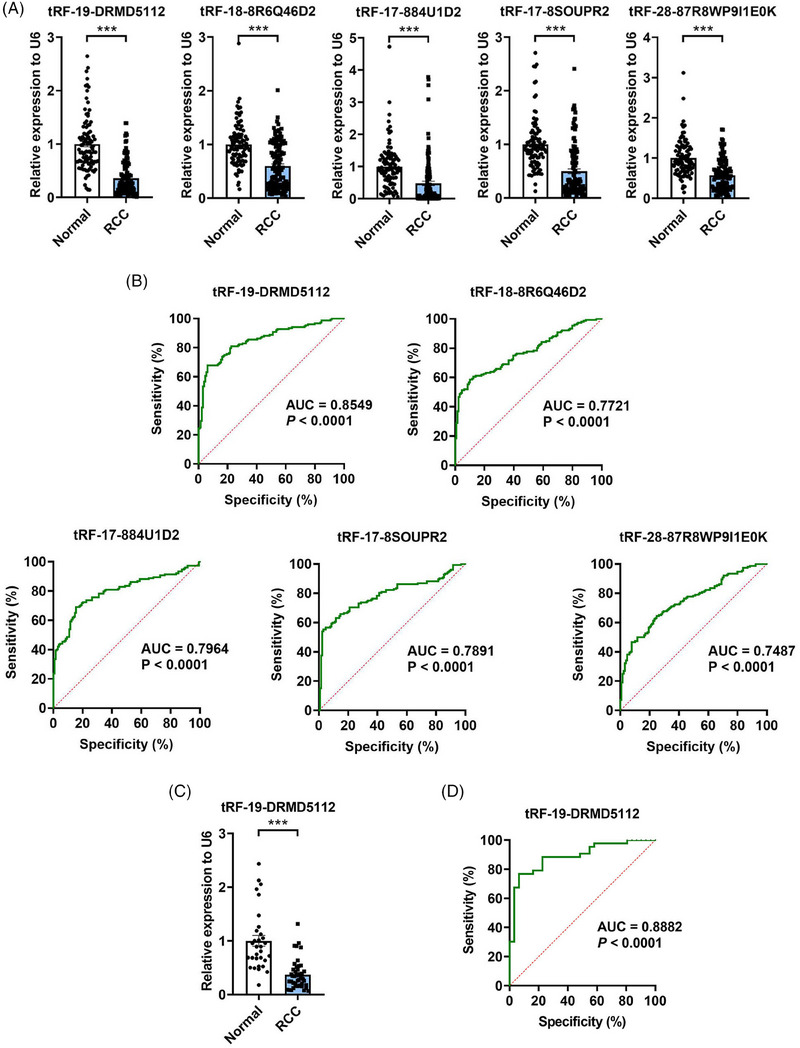
Validation and diagnosis value of differentially expressed plasma tsRNAs in RCC patients. (A) The relative expression levels of 5 plasma tsRNAs in 120 RCC patients and 99 healthy controls using RT‐qPCR. (B) ROC curves for the abilities of 5 individual plasma tsRNAs to discriminate RCC patients from healthy controls in a total of 152 RCC patients and 129 healthy controls. (C) The relative expression levels of tRF‐19‐DRMD5112 in testing set (43 RCC patients and 31 healthy controls) using RT‐qPCR. (D) ROC curve for the ability‐ of plasma tRF‐19‐DRMD5112 level to discriminate RCC patients from healthy controls in testing set. ***p < .001.

We then assessed the clinical usefulness of the five plasma tsRNAs for RCC. By ROC curve analysis, the AUC values for these tsRNAs ranged from 0.7487 to 0.8549 (*p* < .0001; Figure 2B). Among these tsRNAs, tRF‐19‐DRMD5112 showed the best performance (AUC: 0.8549, sensitivity: 80.92%, and specificity: 77.52%; Figure 2B). Furthermore, we assessed the values of these 5 plasma tsRNAs in early‐stage RCC patients (stage I, 118 out of 152 RCC patients) diagnosis and figured out the range of AUC values from 0.7411 to 0.8650 (*p* < .0001; **Figure** **S4**). tRF‐19‐DRMD5112 also showed top performance (AUC: 0.8650, sensitivity: 80.51%, and specificity: 81.31%; **Figure** **S4**), which presented even higher diagnostic value in early‐stage RCC patients compared to that in all RCC patients. To construct a tsRNA‐based diagnostic model, we first performed LASSO‐penalised logistic regression analysis using training and validation sets. Two tsRNAs, tRF‐19‐DRMD5112 and tRF‐18‐8R6Q46D2, were selected for the optimal model construction (**Figure** **S5**). However, in the following multivariate logistic regression analysis, tRF‐19‐DRMD5112 was the only tsRNA independently correlated with RCC (*p* < .001; **Table** **S7**). Taken together, the single expression level of tRF‐19‐DRMD5112 in plasma could be used as the diagnostic model for RCC.

To further assess the diagnostic value of the model, a testing set (31 healthy controls and 43 RCC patients, **Table** **S8**) was utilised for external validation. Consistent with the results above, plasma tRF‐19‐DRMD5112 expression was significantly decreased in RCC patients (*p* < .001; Figure 2C) and could effectively distinguish RCC patients from healthy controls (AUC: 0.8882, sensitivity: 88.37%, and specificity: 77.42%, *p* < .001; Figure 2D).

In addition to the diagnostic value, we further explored the potential function of these 5 tsRNAs in modulating RCC biological behaviours. Expression levels of tRF‐19‐DRMD5112 (17/21, *p* = .0028), tRF‐18‐8R6Q46D2 (13/21, *p* = .0281), and tRF‐17‐8SOUPR2 (17/21, *p* = .0032) expression levels were obviously higher in RCC tissues than that in paired normal tissues, while tRF‐28‐87R8WP9I1E0K (16/21, *p* = .0011) was obviously decreased in RCC tissues (Figure 3A). Characteristics of enrolled RCC patients were listed in **Table** **S8**. Furthermore, we constructed corresponding mimics of the five tsRNAs and transfected them to RCC cells, respectively (**Figure** **S6**). EdU assay, colony formation assay, and sphere formation assay found that only tRF‐28‐87R8WP9I1E0K substantially reduced the proliferation and self‐new abilities of RCC cells (Figure 3B **–D**). Transwell assay and cell scratch test showed that tRF‐28‐87R8WP9I1E0K could markedly decrease the migration ability of RCC cells (Figures 3E and **S7**). Moreover, RCC cells over‐expressed with tRF‐19‐DRMD5112, tRF‐18‐8R6Q46D2, tRF‐17‐8SOUPR2 or tRF‐28‐87R8WP9I1E0K showed reduced invasion ability (Figure 3F). Taken together, these results demonstrated that tRF‐28‐87R8WP9I1E0K played a powerful role in suppressing tumour progression of RCC.

**FIGURE 3:**
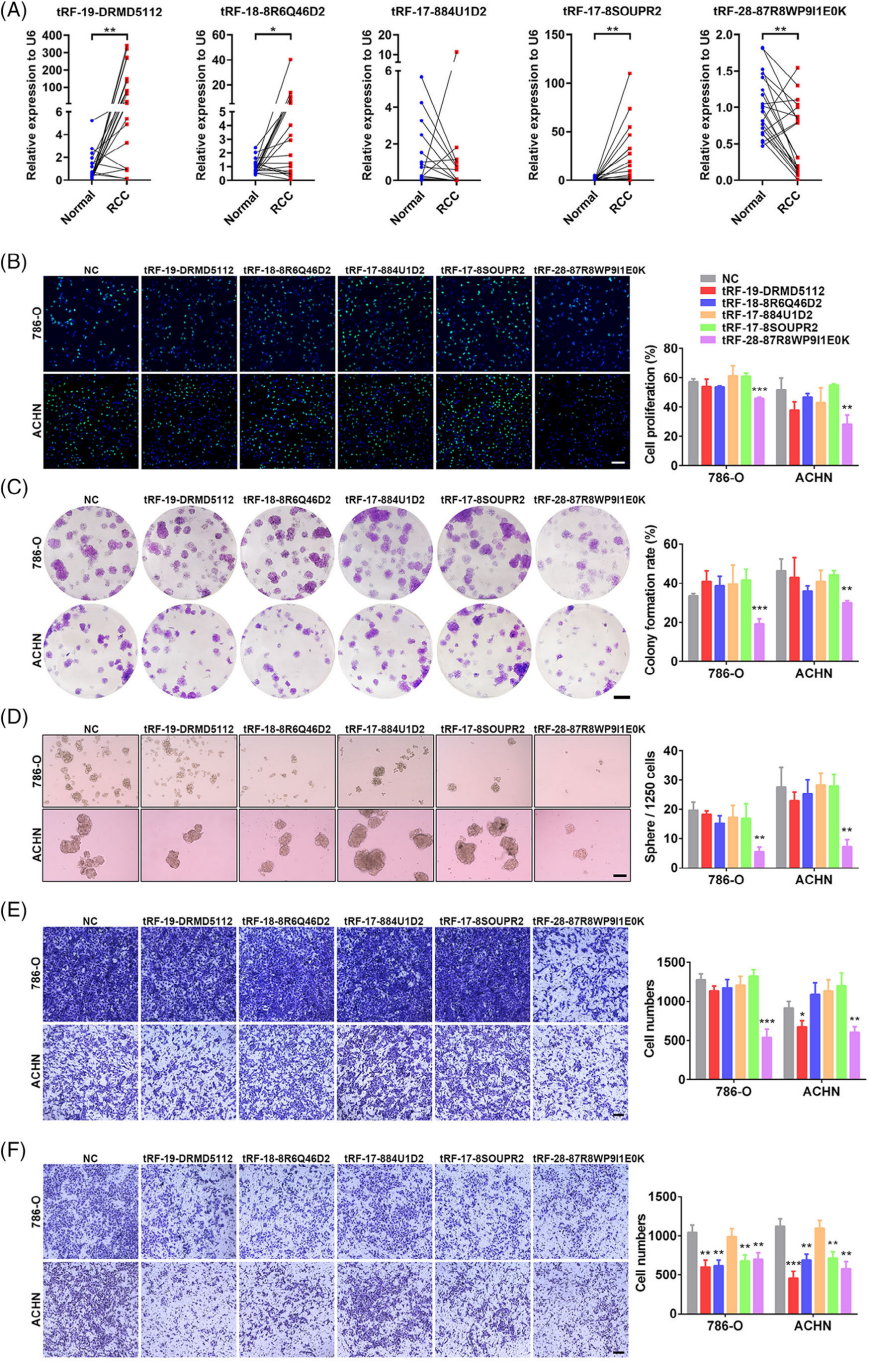
Biology functions of these 5 tsRNAs in RCC. (A) Relative expression levels of the 5 tsRNAs in RCC tissues and paired normal kidney tissues by RT‐qPCR, *N* = 21. (B) Representative images (Left) and histogram statistics (Right) from EdU assay of RCC cells transfected with individual tsRNA mimics or negative controls, scale bar = 50 μm. (C) Representative images (Left) and histogram statistics (Right) from colony formation assay of RCC cells transfected with individual tsRNA mimics or negative controls, scale bar = 0.4 cm. (D) Representative images (Left) and histogram statistics (Right) from sphere formation assay of RCC cells transfected with individual tsRNA mimics or negative controls, scale bar = 100 μm. (E) Representative images (Left) and histogram statistics (Right) from transwell migration assay of RCC cells transfected with individual tsRNA mimics or negative controls, scale bar = 200 μm. (F) Representative images (Left) and histogram statistics (Right) from transwell invasion assay of RCC cells transfected with individual tsRNA mimics or negative controls, scale bar = 200 μm. Cell experiment was repeated three times independently, *p < .05; **p < .01; ***p < .001.

Structural prediction showed that tRF‐28‐87R8WP9I1E0K was located in position 1−28 of tRNA‐Glu‐TTC, which was of the tRF‐5c type (Figure 4A). Considering that tsRNAs might exert the miRNA‐like function, repressing the translation of target mRNAs via binding to AGO proteins and seed sequence‐based canonical target recognition,^5^, ^6^, ^7^ we used targetscan_custom (https://www.targetscan.org/vert_40/seedmatch.html)^8^ and RNAhybrid (https://bibiserv.cebitec.uni‐bielefeld.de/rnahybrid/)^9^ to predict target genes of tRF‐28‐87R8WP9I1E0K (**Table** **S9**). Gene Ontology (GO) analysis of these predicted targets identified multiple enriched GO terms including negative regulation of gene expression, transcription factor activity, protein kinase activity and so forth (Figure 4B, **Table** **S10**). KEGG and WiKi pathway analysis showed that tRF‐28‐87R8WP9I1E0K might be involved in cGMP‐PKG signalling pathway, amphetamine addiction, energy metabolism and other pathways (Figure 4C, **Table** **S10**). Together, these results reminded us that tRF‐28‐87R8WP9I1E0K might modulate the tumour biology of RCC through above mechanisms.

**FIGURE 4:**
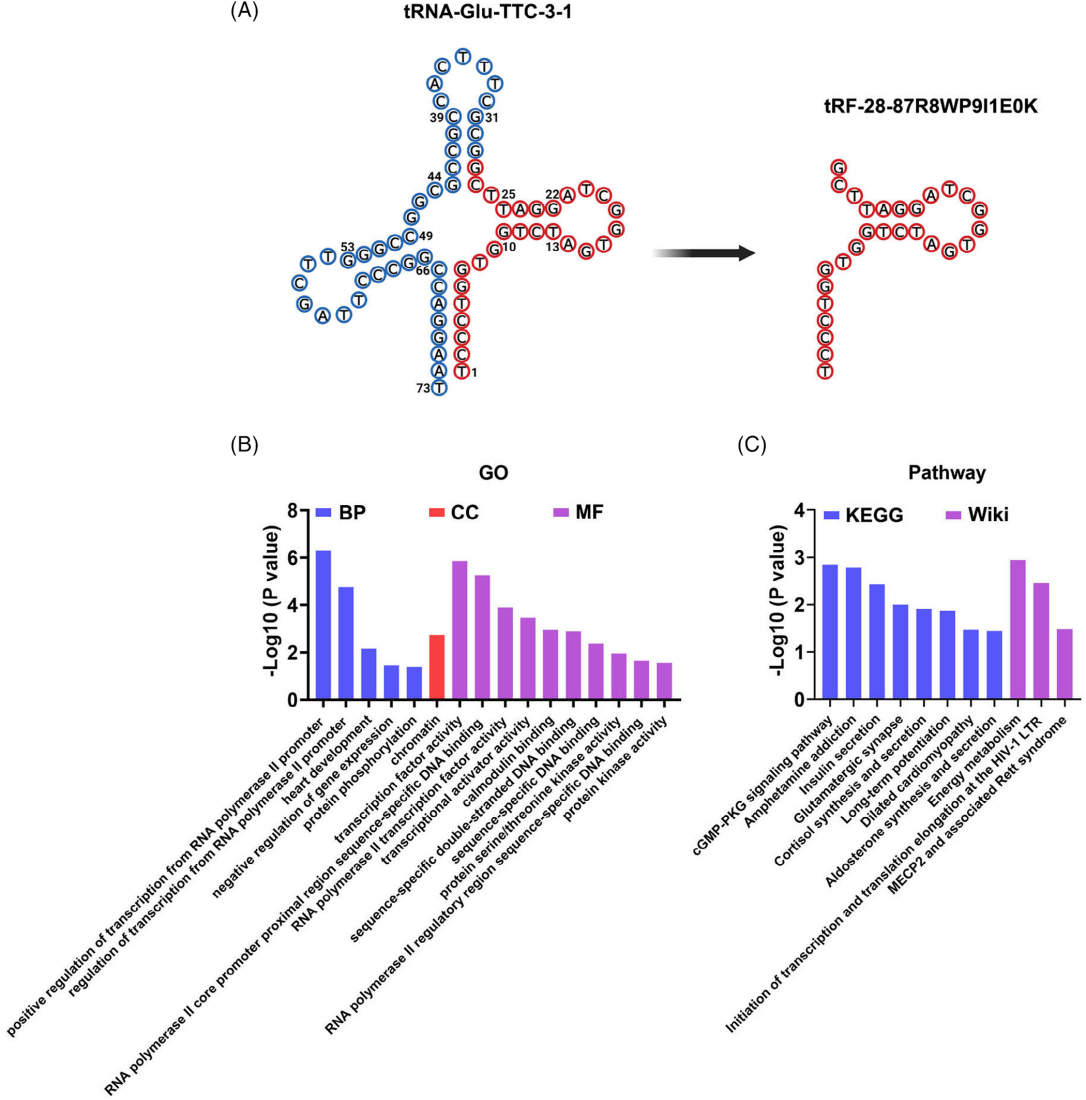
Enrichment analysis of predicted target genes of tRF‐28‐87R8WP9I1E0K. (A) Secondary structure prediction of tRF‐28‐87R8WP9I1E0K. (B) GO analysis of the predicted targets of tRF‐28‐87R8WP9I1E0K. (C) Pathway analysis of the predicted targets of tRF‐28‐87R8WP9I1E0K.

In conclusion, we revealed plasma tsRNA expression patterns in RCC patients for the first time, and identified tRF‐19‐DRMD5112 with favourable diagnostic values for RCC (especially for early stage), providing novel potential biomarker for accurate RCC diagnosis. In addition, tRF‐28‐87R8WP9I1E0K is demonstrated to possess powerful anti‐tumour function, which warrants further exploration to develop a novel tsRNA‐based therapeutic strategy for RCC.

## AUTHOR CONTRIBUTIONS

MD, WD and HG conceived and designed the study. MD, WD and HG wrote the manuscript. MD and WD performed the quantification experiments. MD, WM and XG performed cell experiments. MD, WD and WM performed the bioinformatic analysis and statistical analysis. WZ, WC, XG, YL, CJ and GL contributed to sample collection. All authors read and approved the final manuscript.

## CONFLICT OF INTEREST STATEMENT

The authors declare they have no conflicts of interest.

## FUNDING

This work was supported by grants from the National Natural Science Foundation of China (81902571 to M.D., 82173160 to W.D., and 81972388 to H.G.), Nanjing Medical Science and technology development Foundation (ZKX22024 to M.D.).

## ETHICS STATEMENT

The study was evaluated and authorised by the Ethics Committee of Nanjing Drum Tower Hospital (2021‐582‐01), and all participants voluntarily signed an informed consent.

## Supporting information

Supporting Information

## Data Availability

The datasets used in this study are available upon reasonable request from the corresponding author.
