# Supplementary material for: Plasma tsRNAs as novel diagnostic biomarkers for renal cell carcinoma
Source: Clin Transl Med. 2024 Feb 15;14(2):e1575. [Article in Catalan] doi: 10.1002/ctm2.1575 (PMC10867590; doi:10.1002/ctm2.1575)
Supplement: Supplementary file 1 — Supporting Information [file CTM2-14-e1575-s001.docx]

**Supplementary materials**

**Materials** **and** **methods**

**Study participants and design**

For plasma tsRNA screening and validation, we enrolled a total of 200 patients with pathologically diagnosed RCC from Department of Urology and 165 healthy volunteers from routine physical checks. All participants were recruited at Nanjing Drum Tower Hospital from 2021 Jan to 2023 Dec. The screening set had 10 subjects, including 5 RCC patients and 5 healthy controls. Eligibility criteria for RCC patients were: 1) confirmed diagnosis of RCC; 2) no previous history of renal cancer or other types of cancers; 3) previously without any therapeutic treatments; 4) free of synchronous multiple cancers. Eligibility criteria for healthy controls were: 1) no previous history of cancer; 2) no chronic conditions; 3) normal physical activity level. The training set involved 32 RCC patients and 30 healthy controls, the validation set involved 120 RCC patients and 99 healthy controls, and the testing set involved 43 RCC patients and 31 healthy controls, all with the same eligibility criteria as the screening cohort. The tumor grading and staging were classified according to the WHO Classification for renal carcinoma. For analyzing tsRNAs expression in tumor tissues, we enrolled 21 RCC patients with the above eligibility criteria. The study was evaluated and authorized by the Ethics Committee of Nanjing Drum Tower Hospital (2021-582-01), and all participants voluntarily signed an informed consent.

**Sample collection**

All samples were collected prior to any treatment. Plasma was prepared by centrifuging twice (1500 × g, 20 min, 4 ℃; 3000 × g, 15 min, 4 ℃) and stored at − 80 ℃. In addition, 50 − 100 mg fresh renal cancer tissues and matched normal kidney tissues ( ≥ 2 cm from the resection margin) were collected from RCC patients underwent renal tumor resection. Tissues were washed with saline, frozen in liquid nitrogen and then stored at − 80 ℃.

**Small RNA sequencing and tsRNAs analysis**

Plasma RNA was extracted from 5 RCC patients and 5 healthy controls with TRIzol reagent (Thermo Fisher Scientific, Waltham, MA, USA), respectively. The integrity and quantity of each RNA sample was checked using agarose gel electrophoresis and a Nanodrop™ Spectophotometer. All pre-treatment, library construction and deep sequencing of tRF and tiRNA were performed by Aksomics (Shanghai, China), as previously described in detail.^1^ Sequences of cytoplasmic and mitochondrial tRNAs were obtained from GtRNAdb^2^ and tRNAscan-SE^3^ software, respectively. Differential analysis was performed using negative binomial distribution and Student’s t test. A fold-change threshold was set at ≥ 1.5. *P* < 0.05 was considered significant. tsRNA ID showed in this study were named by MINTbase database (http://cm.jefferson.edu/MINTbase/)^4^.

**Total RNA isolation and quantitative real‑time PCR (RT-qPCR)**

Plasma RNA extraction using acid phenol for RT-qPCR assays was performed as described previously.^5^ For tissues, total RNA was extracted using TRIzol Reagent according to the manufacturer's instructions. The concentrations of RNA were determined by a NanoDrop™ Spectophotometer. For tsRNAs quantification, PrimeScript™ RT reagent Kit (Takara, Shiga, Japan, RR037A) and ChamQ SYBR qPCR Master Mix (Vazyme, Nanjing, China, Q711-02) kit were used for synthesis of cDNA and Real-time PCR, respectively. The detection limits of RT-qPCR assay using tsRNA specific primers were evaluated by the standard curves developed with corresponding synthetic tsRNA oligonucleotides. The expression levels of tsRNAs were normalized to U6 small noncoding RNA.

Plasma tsRNA selection and model construction

To construct a tsRNA-based diagnostic model according to relative expression of plasma tsRNAs to U6 in training and validation sets, we performed LASSO-penalized logistic regression analysis to select the optimal plasma tsRNAs by using the R package “glmnet” based on tenfold cross-validation and lambda.min. During model construction, the Z-score was computed for each selected tsRNA as its relative expression level. Then, the selected plasma tsRNAs were enrolled into a multivariate logistic regression model to calculate their coefficients (β values). If two or more tsRNAs were selected, the plasma tsRNAs-based diagnostic score was constructed using the following formula: diagnostic score = (β_1_×Z-score of tsRNA_1_) + (β_2_×Z-score of tsRNA_2_) + … + (β_n_× Z -score of tsRNA_n_). If only one tsRNA was selected, the relative expression of the tsRNA to U6 in plasma represents the diagnostic model.

**Cells and reagents**

All cells were routinely cultured at a constant temperature of 37°C in 5% CO_2_. Human RCC cell lines 786-O and ACHN were purchased from the Cell Bank of the Chinese Academy of Sciences (Shanghai, China). 786-O and ACHN were maintained in RPMI-1640 medium (Sigma-Aldrich, Darmstadt, Germany) and DMEM medium (Sigma-Aldrich), respectively, both supplemented with 10% fetal bovine serum (FBS, Thermo Fisher Scientific) and 1% penicillin-streptomycin (10,000 U/mL, Thermo Fisher Scientific). Synthetic RNA molecules, including corresponding tsRNA mimics, the scrambled noncoding RNA (ncRNA) and corresponding synthetic tsRNA oligonucleotides were purchased from Generay Biotechnology (Shanghai, China).

**TsRNA transient transfection**

Overexpression of tRF-19-DRMD5112, tRF-18-8R6Q46D2, tRF-17-884U1D2, tRF-17-8SOUPR2, or tRF-28-87R8WP9I1E0K were achieved by transfecting cells with corresponding synthetic RNA mimics and an ncRNA served as a negative control. Briefly, 786-O and ACHN cells were seeded in 6-well plates, and incubated with lipofectamine 2000 (Invitrogen, Carlsbad, CA, USA), ncRNA or corresponding tsRNA mimics (100 pmol/well) according to the manufacturer’s instructions for 24 h. Cells were then collected and subjected to assessment of tumor biological functions.

**EdU (5-ethynyl-2’-deoxyuridine) assay**

786-O and ACHN cells (2 × 10^4^ cells/well) were seeded in 48-well plates and cultured routinely overnight. A BeyoClick™ EdU-488 Cell Proliferation Kit (Beyotime Biotech, Shanghai, China, C0071) was used in EdU assay according to the steps provided by the manufacturer. The cell nucleic were stained with Hoechst. Hoechst and Azide 488 were observed with a fluorescence microscope (Evos FL Auto 2, Invitrogen) and analyzed by ImageJ software.

**Cell colony formation assay**

786-O and ACHN cells (100 cells/well) were seeded in 12-well plates and cultured at 37°C in 5% CO_2_. One week later, unbound cells were removed and the adherent cells were fixed with 4% paraformaldehyde. Subsequently, cells were stained with Crystal Violet Staining Solution (Beyotime Biotech) for 15 minutes. We counted cell colonies containing > 50 cells with a light microscope, and calculated the percentage of formed colonies.

**Sphere formation assay**

786-O and ACHN cells (1250 cells/well) were seeded in ultra-low adhesion 24-well plates, and maintained with corresponding culture median added with 2% B27 (Invitrogen, 17504044), 10 ng/ml FGF-basic (PeproTech, Rocky Hill, NJ, USA, 100-18B), and 20 ng/mL EGF (PeproTech, 100-47). 8 − 10 days later, observation was performed using a microscope (Evos FL Auto 2, Invitrogen) and mammospheres with a diameter > 50 mm were counted.

**Migration and invasion assay**

To evaluate the migration capacity of RCC cells, we performed transwell migration assay and cell scatch test. For transwell migration assay, 786-O and ACHN cells (5 × 10^4^ cells/chamber) were added in the upper transwell chamber (8 μm, Corning Inc, New York, USA.) with FBS-free medium, and the lower well was covered with complete medium. After 18 h, the transwell chambers were collected, fixed and then stained with the Crystal Violet Staining Solution (Beyotime Biotech). Images of the lower surface of the chamber were taken by a microscope (Evos FL Auto 2, Invitrogen). For cell scatch test, 786-O and ACHN cells were seeded in 6-well plates. When cells overgrown the bottom of the wells, we created an artificial wound by using a 200 μL pipette tip. Cells were then maintained with corresponding medium with 2% FBS. Photographs of the wounds were taken by a microscope (AE2000, Motic, Xiamen, Fujian, China) at 0 h and 24 h after the wound was created. For transwell invasion assay, the bottom of the upper chamber was covered with diluted matrigel (BD Biosciences). Then, 786-O and ACHN cells (5 × 10^4^ cells/chamber) resuspended in FBS-free medium were seeded, and the chambers were collected after 24 h.

**Statistical analysis**

All data were analyzed with SPSS V26 and GraphPad Prism V9. Data are presented as the mean ± SD or mean ± SEM. Two-tailed Student’s *t*-test was used to compare differences in variables between two groups. Paired *t*-test was performed to compare tsRNA expression levels between RCC tissues and matched adjacent normal kidney tissues. We used ROC curve analysis to assess the diagnostic values of plasma tsRNAs for RCC, and univariate and multivariate logistic regression analysis to analyze the associations between plasma tsRNAs and RCC. *P* < 0.05 was considered statistically significant.

**Reference**

1. Jin F, Yang L, Wang W, et al. A novel class of tsRNA signatures as biomarkers for diagnosis and prognosis of pancreatic cancer. *Mol Cancer* 2021; 20(1): 95.
2. Chan PP, Lowe TM. GtRNAdb 2.0: an expanded database of transfer RNA genes identified in complete and draft genomes. *Nucleic Acids Res* 2016; 44(D1): D184-9.
3. Lowe TM, Chan PP. tRNAscan-SE On-line: integrating search and context for analysis of transfer RNA genes. *Nucleic Acids Res* 2016; 44(W1): W54-7.
4. Pliatsika V, Loher P, Telonis AG, Rigoutsos I. MINTbase: a framework for the interactive exploration of mitochondrial and nuclear tRNA fragments. *Bioinformatics* 2016; 32(16): 2481-9.
5. Wang C, Ding M, Xia M, et al. A Five-miRNA Panel Identified From a Multicentric Case-control Study Serves as a Novel Diagnostic Tool for Ethnically Diverse Non-small-cell Lung Cancer Patients. *EBioMedicine* 2015; 2(10): 1377-85.

**Supplemental Figures**

**
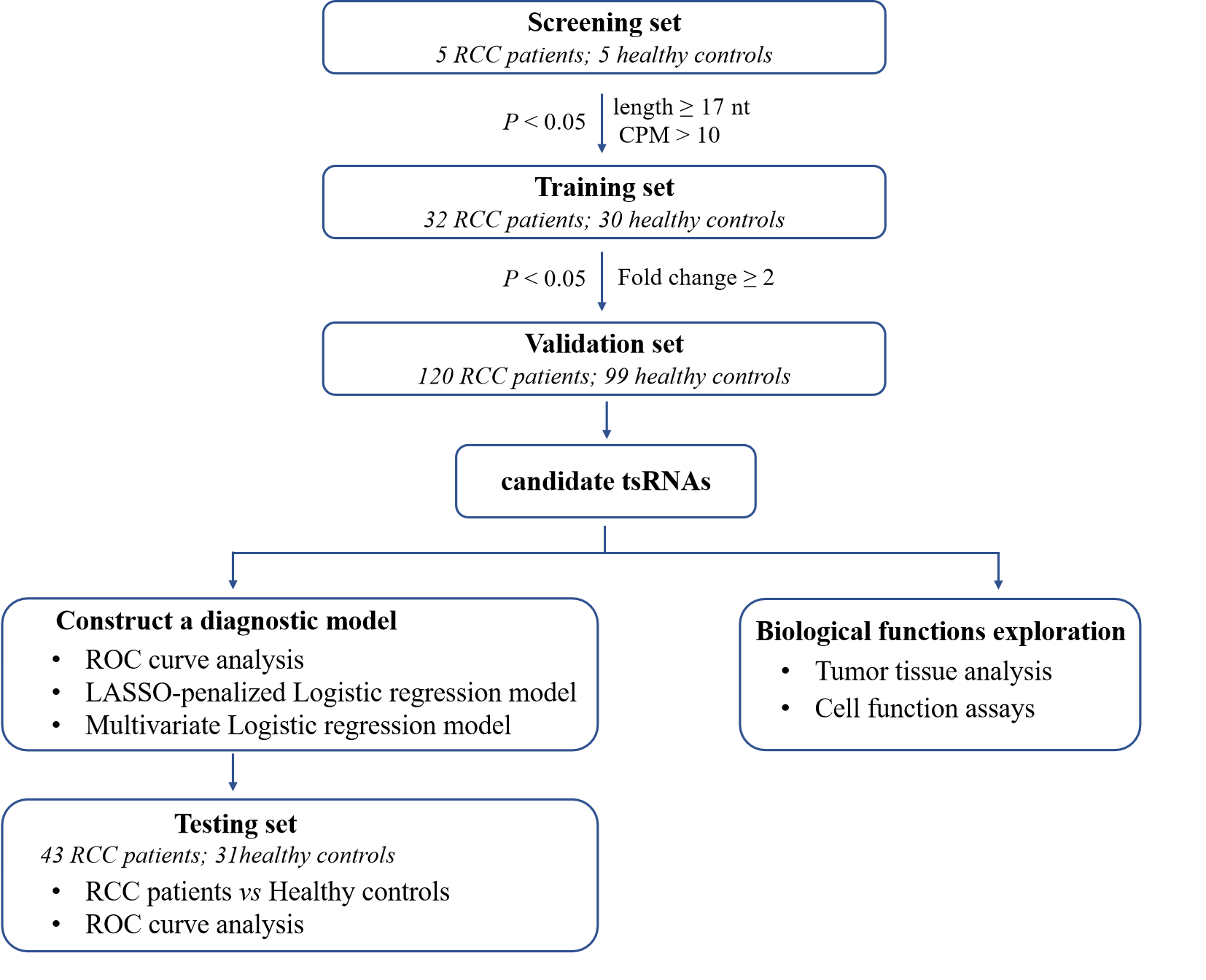
**

**Fig. S1.** The research route diagram.

**
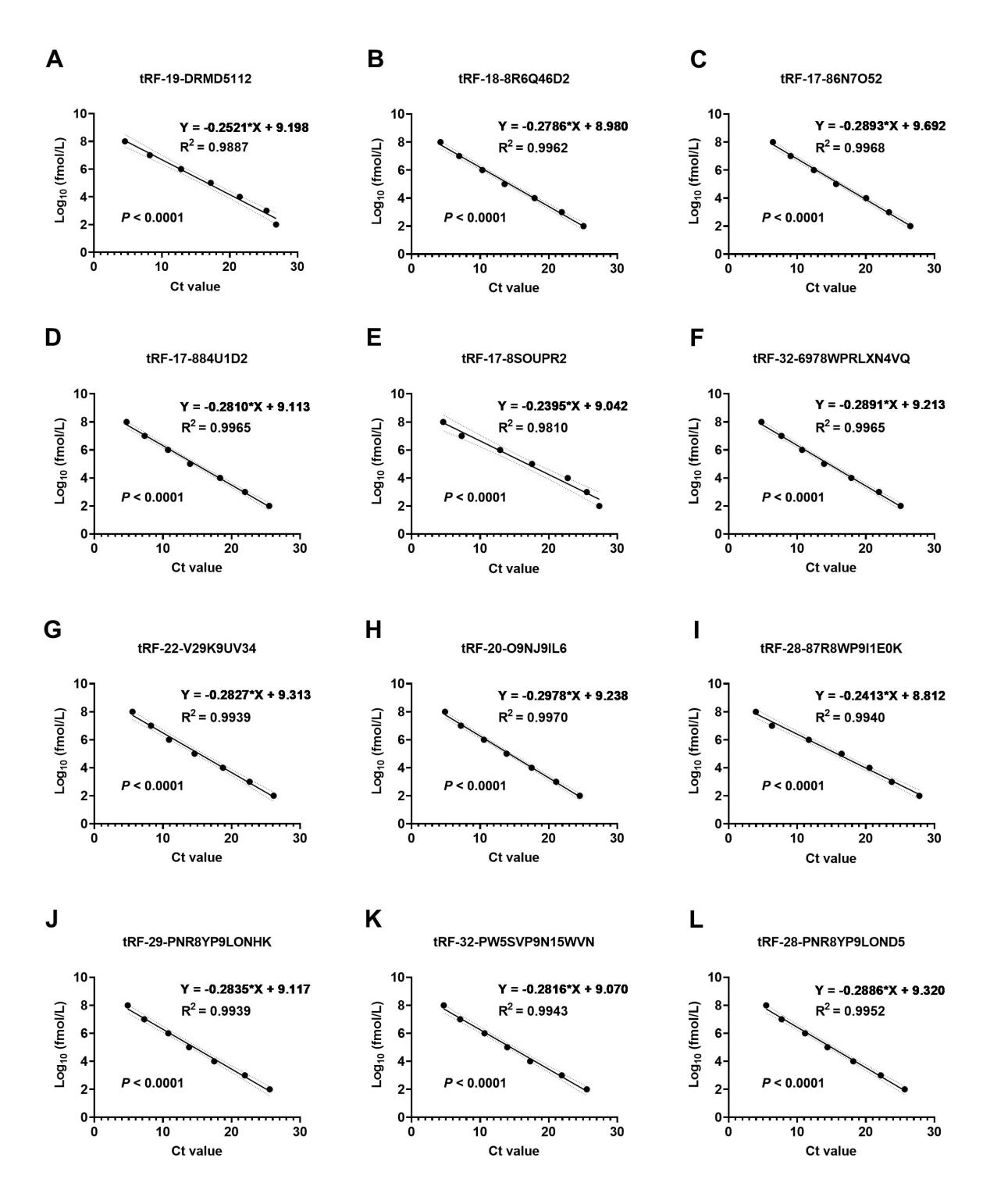
**

**Fig. S2.** The detection limits of tsRNA specific primers in RT-qPCR assay. (A–L) Standard curves of 12 tsRNA specific primers in RT-qPCR assay.

**
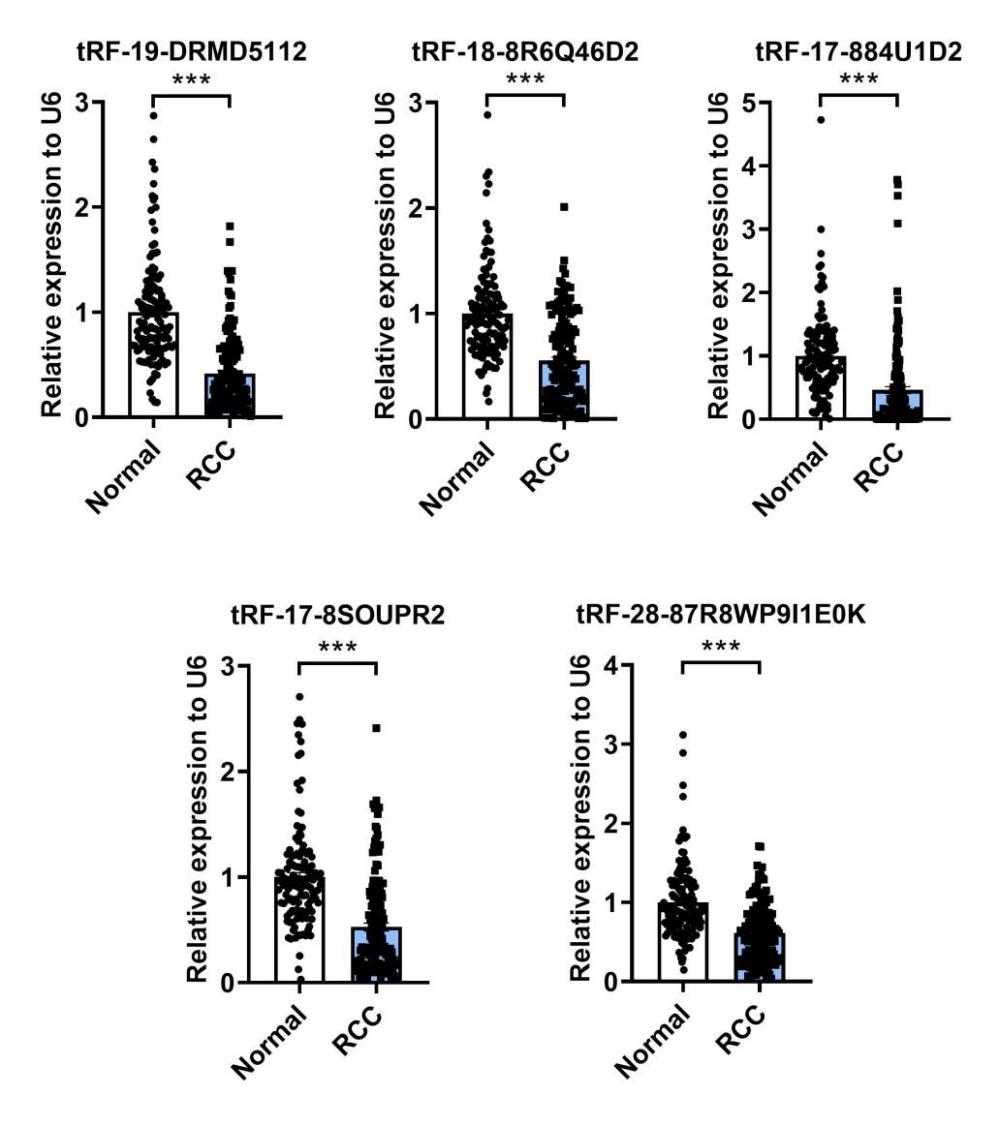
**

**Fig. S3.** Differentially expressed plasma tsRNAs in RCC patients. The relative expression levels of 5 plasma tsRNAs in 152 RCC patients and 129 healthy controls using RT-qPCR. ****P* < 0.001.


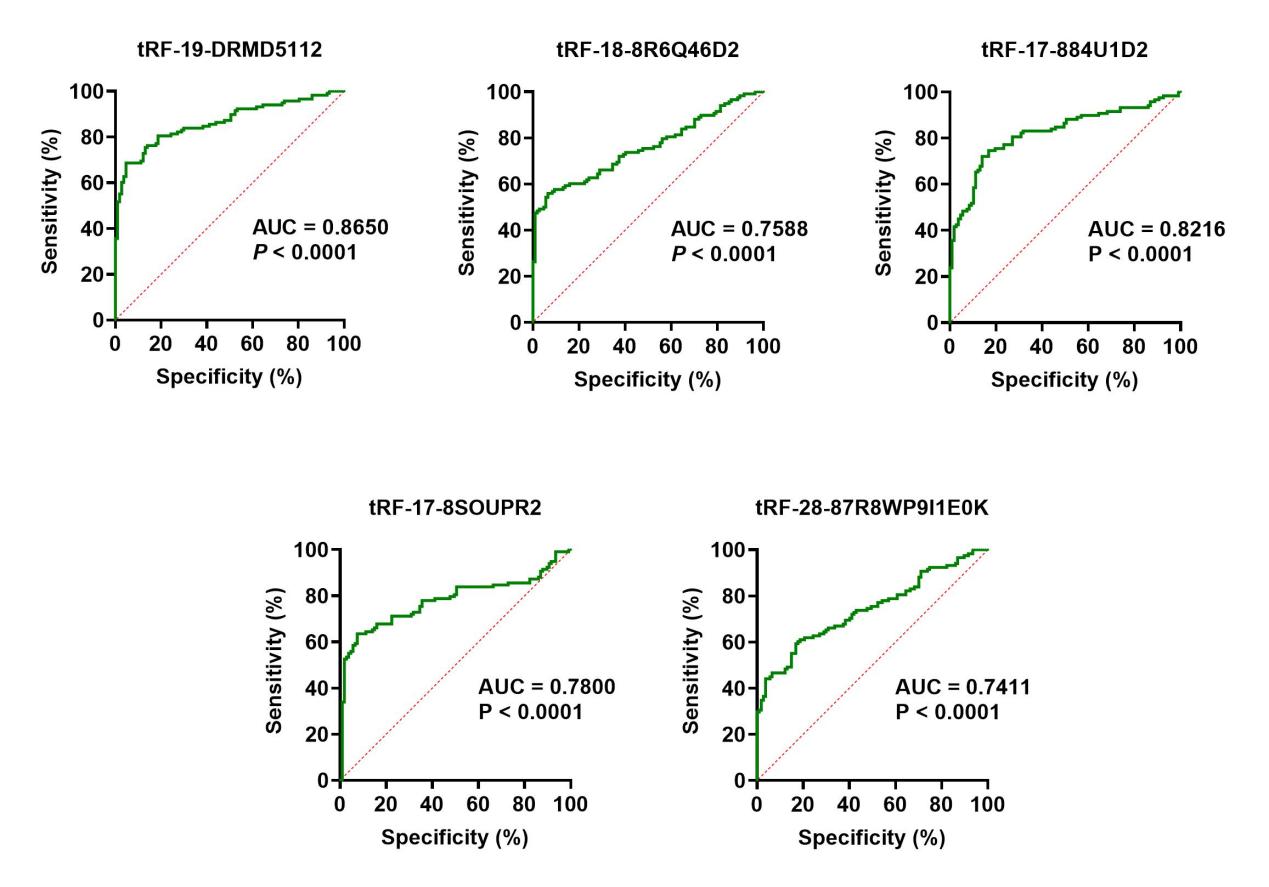


**Fig. S4.** ROC curve analysis of the 5 plasma tsRNAs in early-stage RCC patients. ROC curves for the ability of 5 individual plasma tsRNAs to discriminate early-stage RCC patients (stage I) from healthy controls in a total of 118 RCC patients and 129 healthy controls.

**
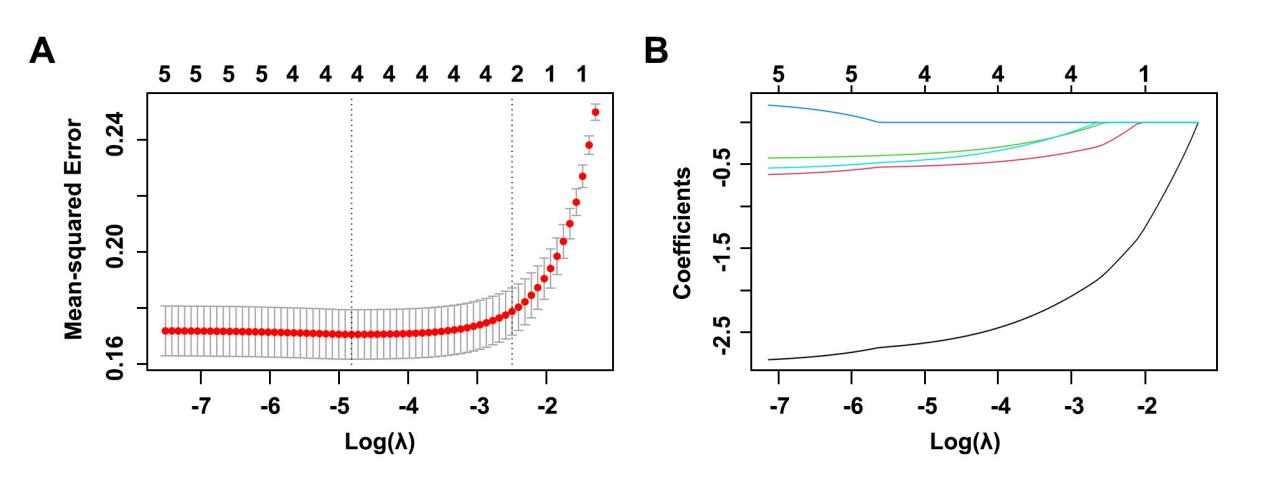
**

**Fig. S5.** Selection of the optimal plasma tsRNAs for modeling by LASSO-penalized logistic regression analysis. (A) The optimal plasma tsRNAs were selected by tenfold cross-validation and lambda.min. (B) LASSO coefficient profile of plasma tsRNAs.

**
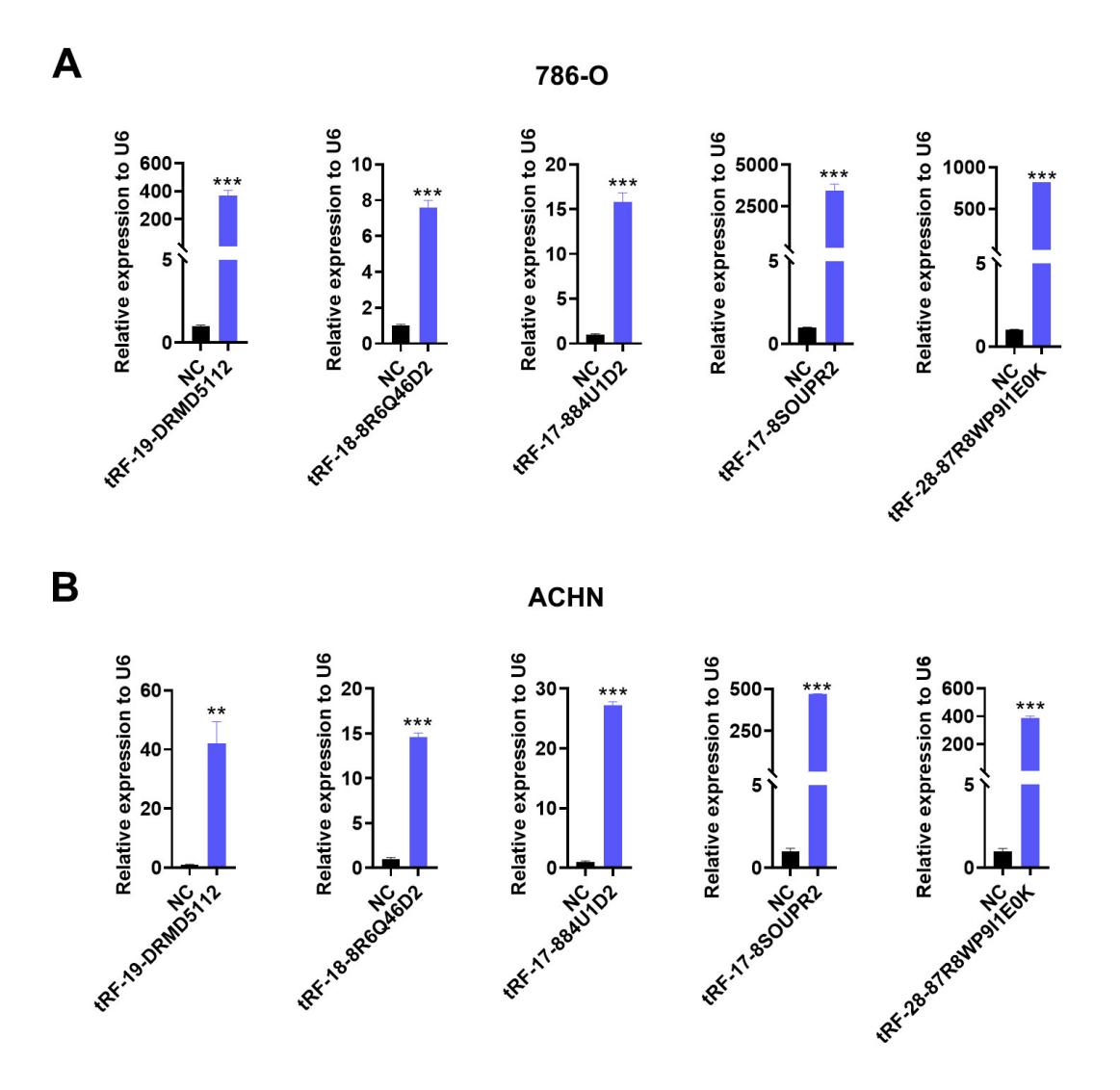
**

**Fig. S6.** The over-expression efficiency of tsRNA mimics in RCC cells. (A) The relative expression levels of tsRNAs in 786-O cells respectively transfected with 5 tsRNA mimics by RT-qPCR assay. (B) The relative expression levels of tsRNAs in ACHN cells respectively transfected with 5 tsRNA mimics by RT-qPCR assay. ***P* < 0.01; ****P* < 0.001.


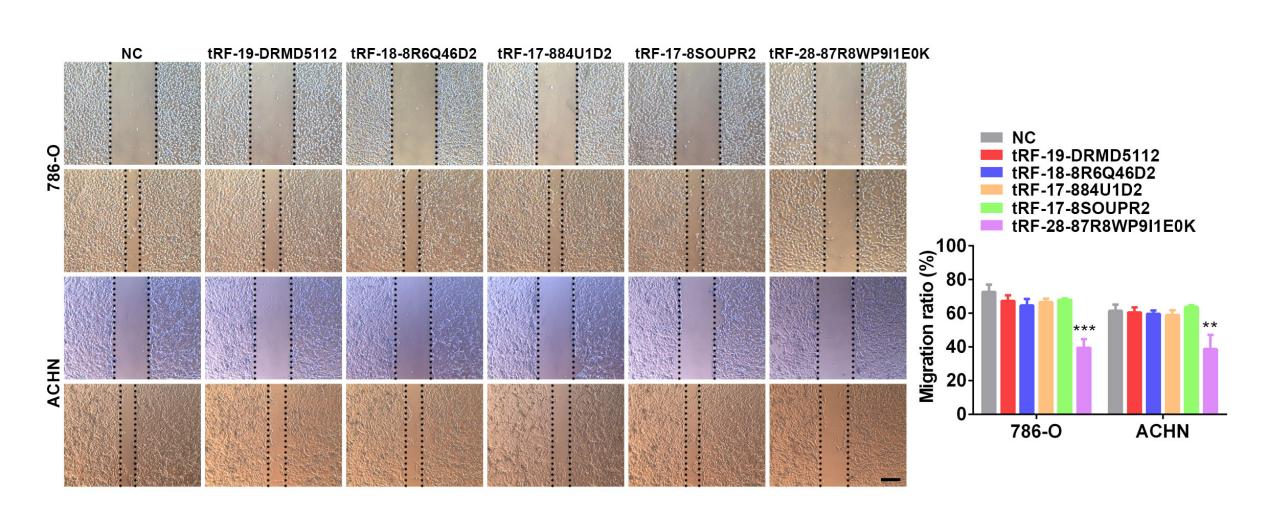


**Fig. S7.** Migration suppressive abilities of the 5 tsRNAs in RCC cells. Representative images (Left) and histogram statistics (Right) from cell scatch test of RCC cells transfected with individual tsRNA mimics or negative controls, scale bar = 500 µm. ***P* < 0.01; ****P* < 0.001.

**Supplemental Tables**

**Table S1** RT-qPCR primer sequences

**Table S2** The 12 significantly differentially expressed tsRNAs in small RNA sequencing

^a^The database ID of MINTbase.

^b^The average of log scaled CPM. The values were calculated by Negative Binomial Generalized Linear Model.

^c^The difference in mean between two groups, RCC_CPM - Normal_CPM.

**Table S3** The relative expression of tsRNAs in plasma from normal controls and RCC patients in the training set

^a^*P* values were derived using two-tailed Student’s t-test to compare values for the two parameters in each category.

**Table S4** The relative expression of tsRNAs in plasma from normal controls and RCC patients in the validation set

^a^*P* values were derived using two-tailed Student’s t-test to compare values for the two parameters in each category.

**Table S5** Demographic and clinical features of the RCC patients and normal controls in the training and validation sets

^a^Age data are presented as the mean (SD).

^b^P values were derived using two-tailed Student’s t-test.

^c^P values were derived using two-sided χ2 test.

^d^The tumor grading and staging were classified according to the WHO Classification for renal carcinoma.

**Table S6** Correlation of five plasma tsRNAs expression to characteristics in RCC patients

^a^*P* values were derived using two-tailed Student’s t-test to compare values for the two parameters in each category.

^b^The tumor grading and staging were classified according to the WHO Classification for renal carcinoma.

**Table S7** Univariate and multivariate logistic regression analyses of plasma tsRNAs for RCC.

**Table S8** Characteristics of RCC patients involved in tumor tissue study

**Table S9** Predicted target genes of tRF-28-87R8WP9I1E0K

| **Target gene** | **Conserved sites in 3'UTRs** | **Target gene** | **Conserved sites in 3'UTRs** | **Target gene** | **Conserved sites in 3'UTRs** |
| --- | --- | --- | --- | --- | --- |
| ATOH8 | 2 | UBASH3B | 1 | DCUN1D3 | 1 |
| LOC57228 | 2 | USP20 | 1 | EDC3 | 1 |
| MECP2 | 2 | VMAC | 1 | EI24 | 1 |
| ABL2 | 1 | ZNF282 | 1 | EMID2 | 1 |
| ACSL4 | 1 | ZNF414 | 1 | ENAH | 1 |
| ADAMTS4 | 1 | ZNF609 | 1 | EPB41L5 | 1 |
| ADD2 | 1 | PTPRT | 2 | EPHA7 | 1 |
| ANKHD1-EIF4EBP3 | 1 | ANGEL1 | 1 | ESRRA | 1 |
| APBA1 | 1 | API5 | 1 | FAM152B | 1 |
| ARAF | 1 | ARIH2 | 1 | FAM80A | 1 |
| ARHGEF2 | 1 | ATP1B4 | 1 | FBXL19 | 1 |
| ARL8A | 1 | C14orf43 | 1 | FOSL2 | 1 |
| ASTN1 | 1 | C1orf109 | 1 | FUT10 | 1 |
| BAHCC1 | 1 | C5orf41 | 1 | GAB2 | 1 |
| C1orf220 | 1 | CD226 | 1 | GIGYF1 | 1 |
| C9orf19 | 1 | DENND3 | 1 | GIT2 | 1 |
| C9orf25 | 1 | DOCK3 | 1 | IGSF9B | 1 |
| CACNB3 | 1 | DTX1 | 1 | IL28RA | 1 |
| CALU | 1 | EIF5A2 | 1 | INSR | 1 |
| CD248 | 1 | FAM100B | 1 | KANK4 | 1 |
| CDK5R2 | 1 | FBXO41 | 1 | KCNE1L | 1 |
| CLCN5 | 1 | FIZ1 | 1 | KIAA0100 | 1 |
| CNNM1 | 1 | FREM2 | 1 | KLHDC7A | 1 |
| COTL1 | 1 | FXR2 | 1 | LDB1 | 1 |
| CTDSPL | 1 | GPC4 | 1 | LL22NC03-75B3.6 | 1 |
| DAAM2 | 1 | GPR3 | 1 | LPCAT3 | 1 |
| DNAJB1 | 1 | HTT | 1 | LRRC58 | 1 |
| EIF4EBP3 | 1 | ICA1L | 1 | MAML1 | 1 |
| FLJ25404 | 1 | IKZF4 | 1 | MAPK11 | 1 |
| GATAD2B | 1 | KCNK13 | 1 | MARCH4 | 1 |
| GJC1 | 1 | KLHL18 | 1 | NEO1 | 1 |
| GNAI3 | 1 | LPIN2 | 1 | NFE2L1 | 1 |
| GRAMD1A | 1 | N4BP1 | 1 | NRXN2 | 1 |
| HIPK2 | 1 | NFIB | 1 | NUDCD3 | 1 |
| HOMEZ | 1 | NKIRAS2 | 1 | ORAI3 | 1 |
| IL10 | 1 | NR2C2 | 1 | OTX1 | 1 |
| LIMK2 | 1 | PCDH19 | 1 | P4HA1 | 1 |
| LRP3 | 1 | PGM2L1 | 1 | PAX5 | 1 |
| LRWD1 | 1 | PPP1R9B | 1 | PBX1 | 1 |
| MAPKAPK3 | 1 | SLC12A6 | 1 | PDCD4 | 1 |
| MARCKS | 1 | SLC16A3 | 1 | PI4KB | 1 |
| MED1 | 1 | SOCS1 | 1 | POU3F2 | 1 |
| MGA | 1 | SOX21 | 1 | PPP3R1 | 1 |
| MLL2 | 1 | SP1 | 1 | PTGIS | 1 |
| NME6 | 1 | TNK2 | 1 | PXMP4 | 1 |
| OLA1 | 1 | VCP | 1 | RAB15 | 1 |
| OTP | 1 | WDR22 | 1 | RCE1 | 1 |
| PAPPA | 1 | WDR55 | 1 | SFRS2 | 1 |
| PHOSPHO1 | 1 | WNK4 | 1 | SH3BGRL2 | 1 |
| PIK3IP1 | 1 | WNT4 | 1 | SKAP1 | 1 |
| PKM2 | 1 | ZNF629 | 1 | SLC23A2 | 1 |
| PLOD1 | 1 | ZNF652 | 1 | SMOC1 | 1 |
| PPP2R3A | 1 | ACAN | 1 | SPCS1 | 1 |
| PRCC | 1 | ACVRL1 | 1 | SRF | 1 |
| PRPF40A | 1 | ADAM9 | 1 | SRGAP2 | 1 |
| RBM24 | 1 | ADIPOR2 | 1 | SSR3 | 1 |
| RNF41 | 1 | ADM2 | 1 | STEAP3 | 1 |
| SETD7 | 1 | BCL2L1 | 1 | TANC2 | 1 |
| SIX6 | 1 | BRCC3 | 1 | THAP11 | 1 |
| SKAP2 | 1 | BTNL3 | 1 | TMEM127 | 1 |
| SLC7A8 | 1 | C4orf19 | 1 | TMEM35 | 1 |
| SMG1 | 1 | C7orf51 | 1 | TNPO1 | 1 |
| ST8SIA2 | 1 | CACNA1C | 1 | TNPO3 | 1 |
| SYVN1 | 1 | CCDC120 | 1 | TXLNA | 1 |
| TAF8 | 1 | CCDC49 | 1 | VASP | 1 |
| TMEM32 | 1 | CENTG2 | 1 | WDR47 | 1 |
| TP53 | 1 | CLIP3 | 1 | WDR51A | 1 |
| TPM4 | 1 | CNIH2 | 1 | XPO4 | 1 |
| TPPP2 | 1 | CTSB | 1 | ZFYVE1 | 1 |
| TSGA14 | 1 | DAB2IP | 1 | tcag7.1228 | 1 |

**Table S10** Enrichment analysis of predicted target genes of tRF-28-87R8WP9I1E0K
